# Pulsed electromagnetic fields preconditioned extracellular vesicles derived from mesenchymal stromal cells prevents necroptosis of osteoblasts in osteonecrosis of the femoral head rats

**DOI:** 10.3389/fbioe.2025.1655579

**Published:** 2025-10-09

**Authors:** Xiao-Na Xiang, Jiang-Yin Zhang, Xiang-Xiu Wang, Hong-Chen He, Cheng-Qi He

**Affiliations:** ^1^ Rehabilitation Medicine Center and Institute of Rehabilitation Medicine, West China Hospital, Sichuan University, Chengdu, China; ^2^ Key Laboratory of Rehabilitation Medicine in Sichuan Province, West China Hospital, Sichuan University, Chengdu, China; ^3^ School of Rehabilitation Sciences, West China School of Medicine, Sichuan University, Chengdu, China

**Keywords:** osteonecrosis of the femoral head, extracellular vesicles, bone marrow mesenchymalstromal cells, pulsed electromagnetic fields, necroptosis

## Abstract

**Background:**

Osteonecrosis of the femoral head (ONFH) is a refractory orthopedic disease in which steroids may induce bone cell necroptosis. Extracellular vesicles derived from bone marrow mesenchymal stromal cells (BMSC-EVs) are recognized as novel therapies to improve ONFH. Pulsed electromagnetic fields (PEMFs) increase the paracrine activity of BMSCs. Nonetheless, the effect and mechanism of PEMFs preconditioned BMSC-EVs (BMSC-EVs^PEMFs^) for treating ONFH are unclear.

**Methods:**

The BMSC-EVs^PEMFs^ with different magnetic amplitudes were incubated with dexamethasone-induced MC3T3-E1 cells and the osteogenic differentiation and necroptosis were observed. Furthermore, RNA sequencing of MC3T3-E1 cells incubated with incubated with PEMFs of a specific amplitude or without PEMFs was conducted to identify potential mechanisms involved. Reverse transcription‒quantitative polymerase chain reaction (RT-qPCR), immunofluorescence and Western blotting were performed to detect necroptosis-related pathways. SD rats receiving steroid injections were randomly assigned to receive PBS, BMSC-EVs or BMSC-EVs^PEMFs^ therapy. Micro-CT scan, histological, and immunohistochemical analyses were used to evaluate the therapeutic effects on bone formation and necroptosis of the femoral head in ONFH animals.

**Results:**

The characteristics of the BMSC-EVs^PEMFs^ were similar to those of the BMSC-EVs. *In vitro*, co-culture of osteoblasts and PEMFs with 3 millitesla (mT) amplitude preconditioned BMSC-EVs (BMSC-EVs^PEMFs (3 mT)^ promoted osteogenic differentiation and inhibited cell death. The results of RNA sequencing revealed that the expression of Ripk3 was significantly lower in the BMSC-EVs^PEMFs (3 mT)^ group than in the BMSC-EVs group. RT-qPCR, immunofluorescence and Western blotting revealed that the expression of necroptosis-related molecules (RIPK1, RIPK3, and MLKL) was suppressed in BMSC-EVs^PEMFs (3 mT)^ group (*p* < 0.05). *In vivo*, the BMSC-EVs^PEMFs (3 mT)^ group presented better bone morphology of the femoral head via micro-CT, with a lower protein expression of MLKL and a higher expression of RUNX2 (*p* < 0.05) at 2 weeks, while lower expressions of RIPK1 and RIPK3, and higher levels of RUNX2 and OCN (*p* < 0.05) at the femoral head at 6 weeks after injection than did the BMSCs-EVs group.

**Conclusion:**

PEMFs with 3 mT amplitude preconditioned BMSC-EVs could promote bone formation by inhibiting osteoblasts necroptosis via Ripk1–Ripk3–Mlkl signaling in ONFH.

## Graphical Abstract

**Figure:**
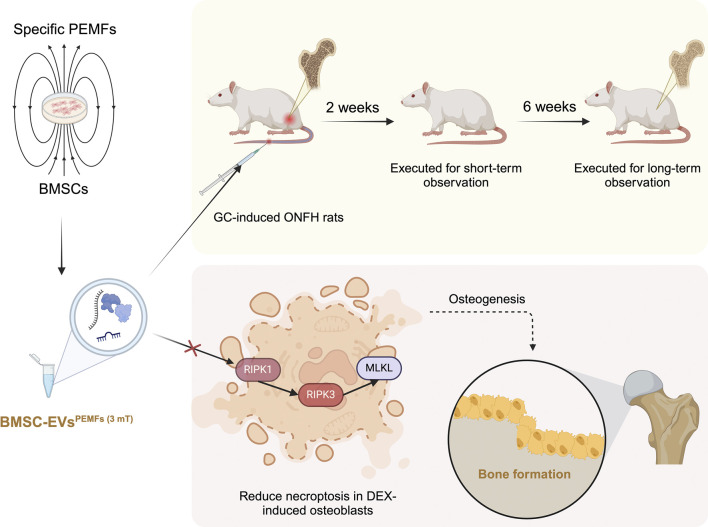

## 1 Introduction

Glucocorticoids, although widely prescribed as anti-inflammatory and immunomodulatory agents, are a major cause of osteonecrosis of the femoral head (ONFH) due to their detrimental effects on bone metabolism (Padhye et al., 2016; Mimura et al., 2023). ONFH affects over 8 million individuals in China, with an average onset age of 58.3 years (Mi crosurgery Department of the Orthopedics Branch of the Chinese Medical Doctor A et al., 2017; Lamb et al., 2019), and most individuals with ONFH suffer pain and collapse of the femoral head leading to subsequent deterioration of the hip joint (Kawano et al., 2020). Since patients are young, guidelines suggest the implementation of multiple techniques aimed at preserving hips (Hannon et al., 2023; Zhao et al., 2020), yet the optimal surgical approach remains debated (Miladi et al., 2018; Sadile et al., 2016; Migliorini et al., 2021). ONFH is characterized by the decrease in bone formation and situ death of bone cells, making it difficult for affected bones to recover (Chan et al., 2020). Numerous studies have reported that imbalanced programmed cell death, such as apoptosis (Zhao et al., 2023; Chen et al., 2020), necroptosis (Fan et al., 2022; Feng et al., 2023) and pyroptosis (Fang et al., 2024), disrupts bone homeostasis and causes osteonecrosis (Shao et al., 2024). Therefore, alternative approaches for preventing bone cell death and the progression of ONFH during the initial phase are urgently needed.

Bone marrow mesenchymal stromal cells (BMSCs) exhibit therapeutic effects for ONFH as their potential to promote osteogenesis and angiogenesis (Daltro et al., 2015). However, challenges such as limited progenitor cell availability, poor survival of transplanted cells, immune rejection, and possible pro-tumor risks restrict their clinical translation (McKinley et al., 2023). Extracellular vesicles (EVs), nanoscale mediators of intercellular communication, have recently attracted attention as cell-free alternatives (Boulestreau et al., 2021; Lu et al., 2022) and new tools for managing diseases (Han et al., 2024; Wang et al., 2025; Liu et al., 2024). BMSC-derived EVs (BMSC-EVs) have been shown to alleviate ONFH by enhancing cell survival (Huang et al., 2020), promoting osteoblast proliferation (Liao et al., 2019), and stimulating bone microvascular endothelial activity (Li L. et al., 2020). Due to the limited accessibility and poor yield of BMSC-EVs (Debbi et al., 2022; Zhou et al., 2024), it is crucial to enhance their specific biological functions.

Preconditioning strategies offer a potential solution. Pulsed electromagnetic fields (PEMFs) are known to regulate MSC proliferation, differentiation, and paracrine activity (Celik et al., 2021; Parate et al., 2020). Recent study indicated that PEMFs regulated the bioactivity of M2 macrophage-derived EVs on decreasing osteoclastogenesis (Trentini et al., 2024). Our previous work further showed that PEMF preconditioning enhances the anti-apoptotic effects of MSC-EVs, with 75 Hz yielding the most pronounced benefits (Xu et al., 2022). Nevertheless, the optimal remain undefined, and the therapeutic efficacy and mechanisms of PEMFs preconditioned BMSC-EVs (BMSC-EVs^PEMFs^) in the ONFH animals are still unclear.

In the present study, we examined the effects of BMSC-EVs^PEMFs^ on osteogenesis and necroptosis, offering an initial exploration of the mechanisms involved. Additionally, we explored the potential of BMSC-EVs^PEMFs^ as an innovative biomimetic approach to enhance bone regeneration and reduce programmed cell death in a model of ONFH rats.

## 2 Materials and methods

### 2.1 Isolation and identification of BMSCs

Three-week-old male Sprague-Dawley rats were humanely euthanized, and the femurs and tibias were harvested under sterile conditions. Culture method was previously reported (Aghaloo et al., 2010; Wu et al., 2024), and operation flow is shown in Supplementary Figure S1. The cells from the third or fourth passage were used for the subsequent experiments.

The levels of cell surface markers, including CD44, CD34, CD45, and CD90, were analyzed following the guidelines provided by the manufacturer (Shi et al., 2014). Additionally, the capacity for multi-lineage differentiation was assessed by Alizarin red staining (ARS), Oil red O staining, and Alcian blue staining (Cyagen, China) after 7-day, 21-day, and 28-day stimulations. Colony-forming unit assays were initially performed with 1 × 10^3^ single-cell suspensions seeded in a 10 cm diameter culture dish (Corning, United States), and reflected by toluidine blue (Beyotime, China) after 14 days.

### 2.2 PEMFs intervention

The PEMFs device (School of Manufacturing Science and Engineering, Sichuan University, China) (Huang et al., 2022; Wang et al., 2022) consists of a pulse generator, a stepper motor driver, and a Helmholtz coil, and produces spatially homogeneous, time-varying magnetic fields in the incubator (seen in Supplementary Figure S2; Supplementary Table S1). There are two trays inside the circular Helmholtz coil chamber, and the output waveform from the signal generator produced a pulsed burst with a duty ratio of 50% (burst width: 6.67 ms; pulse width: 6.67 ms), repeated at a frequency of 75 Hz. The magnetic flux density increased to a predetermined maximal level within approximately 50 μs (with a rise rate of ∼17 T/s) when driving field amplitudes ranged between 0.5 and 3.8 mT. This was measured using a hand-held Gaussmeter (HT201, Hengtong, China). According to previous studies, BMSCs at passage 4 hungered with 5% EVs-free FBS for 48 h, and then were placed in the incubator under PEMFs with 0 (negative control), 1 mT (Celik et al., 2021), 1.6 mT (Huang et al., 2021; Yang et al., 2018), or 3 mT (Parate et al., 2020) amplitudes for 60 min.

### 2.3 EVs isolation, labeling, and uptake

BMSC-EVs were isolated via ultra-centrifugation method as previously described (Guo et al., 2017; Xu et al., 2021) and flow is shown in Supplementary Figure S3. The culture media were harvested, centrifuged at 300 × *g* and 2,000 × *g* to remove debris, and then filtered through a 0.22 μm filter (Merck-Millipore). The supernatant was aliquoted into 15 mL Amicon Ultra-15 devices with a 100 kDa membrane and subjected to centrifugation at 4,000 × *g*. Then, it was ultra-centrifuged at 100,000 × *g* for 70 min (SW32Ti, Beckman Coulter), washed with PBS, and ultra-centrifuged again at the same speed for 70 min. The EVs were carefully resuspended in sterile PBS and stored at −80 °C for subsequent experiments. Transmission electron microscopy (TEM) (JEM-1400FLASH, Japan) was used for observing morphology operating at 80–120 kV. For particle size and number analysis, nanoparticle trafficking analysis (NTA) was completed using the ZetaView system (Particle Metrix, Germany) following the manufacturer’s instructions. Western blot was employed to confirm the presence of markers, such as positive expression of TSG101, CD81, and CD9, and negative expression of Calnexin.

Next process involved incubating EVs with DIO (Beyotime, China) for 15 min at room temperature. After washing with PBS and centrifuging at 100,000 × *g* for 70 min, the various BMSC-EVs were suspended in basal medium (10^10^/mL) and incubated with MC3T3-E1 cells for 48 h at 37 °C (Wu et al., 2019; Li et al., 2022). Then stained by 0.1 g/mL DAPI (Beyotime, China) for 5 min, the MC3T3-E1 cells were placed under a confocal system of high-content screening (PE/Opera Phenix Plus, PerkinElmer, United States) for image capture.

### 2.4 Cell culture and dexamethasone stimulation

MC3T3-E1 osteoblastic cells were purchased from CCTCC (Subclone 14, GDC0188, China) and cultured in α-MEM supplemented with 10% FBS (Lonsera, Uruguay) and 1% penicillin-streptomycin in humidified incubators at 37 °C and 5% CO_2_. Cells were treated with 10 μM DEX (TargetMol, China) *in vitro* to mimic the disease for 24 h (Tao et al., 2017). BMSC-EVs, BMSC-EVs^PEMFs (1 mT)^, BMSC-EVs^PEMFs (1.6 mT)^ and BMSC-EVs^PEMFs (3 mT)^ at a dosage of 10^10^/mL were co-cultured with MC3T3-E1 osteoblastic cells for 48 h.

### 2.5 Annexin V staining

Annexin V-FITC/PI apoptosis detection kit (Cell Signaling Technology, Danvers, MA, United States) was used for distinguishing between live, early apoptotic, and late apoptotic/necrotic cells (Zhu et al., 2021). Then the results were obtained using a flow cytometer (FACSAria III, BD, United States).

### 2.6 Alkaline phosphatase (ALP) and alizarin red staining (ARS) staining

Following osteogenic induction for periods of 7 and 21 days, the cells were fixed with a solution of 4% paraformaldehyde (Biosharp, China). Subsequently, alkaline phosphatase (ALP) activity was assessed with a BCIP/NBT staining kit (Beyotime, China). To evaluate the formation of mineralized nodules, Alizarin Red S (ARS) staining (Beyotime, China) was conducted for 20 min. The cells were then examined microscopically (Ti2, Nikon, United States) to assess osteogenic differentiation.

### 2.7 Western blotting analysis

Total proteins were extracted and examined using a protein extraction kit (Beyotime, China) and BCA method with a commercial kit (Thermo Fisher Scientific, United States) following established protocols. Then the proteins were separated using 10% SDS-PAGE (EpiZyme, China) and were then transferred onto polyvinylidene fluoride membranes with a pore size of 0.22 μm. GAPDH was used for normalization. The experiments were performed in triplicate. The information concerning the antibodies used and their concentrations is presented in Supplementary Table S2.

### 2.8 Reverse transcription-quantitative polymerase chain reaction (RT-qPCR)

Total RNA was extracted with TRIzol reagent (Takara, Japan) from femoral heads and cultured cells, followed by reverse transcription to generate the first-strand cDNA using the Stand cDNA Synthesis SuperMix for qPCR Kit (Hifair III, Yeasen, China). PCR was conducted with the SYBR Green PCR master mix (HieffUNICON, Yeasen, China) utilizing a Bio-Rad CFX Connect real-time system (Bio-Rad, United States). Primer sequences are shown in Supplementary Table S3 and Supplementary Table S4. The experiments were conducted in three replicates, and the data were analyzed by the 2^−ΔΔCT^ method (Bernáldez et al., 2017).

### 2.9 Immunofluorescence analysis

Briefly, after being fixed, permeabilization and blocked, cells were incubated with primary antibodies: RUNX2, CON, RIPK1 or RIPK3 (1:200) (Beyotime, China). After being washed twice, the 96-well (PerkinElmer, United States) were subsequently treated with secondary goat anti-rabbit antibody (Alexa Fluor 488, Beyotime, China) at 37 °C for 1 h. F-action and nuclei were co-stained for 20 min with phalloidin (Actin-Tracker Red-594, Beyotime, China) and for 5 min with DAPI, respectively.

### 2.10 RNA sequencing analysis

MC3T3-E1 cells treated with DEX or with proper EVs were compared. The sequence and filtering of clean reads were completed as previously described (Thompson et al., 2020). A cDNA library was generated using pooled RNA from two groups and sequenced utilizing the Illumina Novaseq™ 6000 sequencing platform (LC-Biotechnology CO., Ltd., Hangzhou, China) (Kim et al., 2019; Kovaka et al., 2019). The raw sequence data have been submitted to the NCBI Short Read Archive (SRA) with the access number PRJNA1115973 (SAMN41518211-SAMN41518218). DESeq2 soft were utilized for analyzing the differentially expressed genes (DEGs). A volcano plot was performed by DEGs (Fold change ≥ 1.1 and false discovery rate (FDR) < 0.1). DEGs were included for further functional analysis based on GO and Kyoto Encyclopedia of Genes and Genomes (KEGG) databases.

### 2.11 Animal experiments

Approval for the animal experiments in this study was obtained from the Animal Ethical Committee. According to the guidelines for sample size calculations of Boston University and the study of Li and colleagues (Li et al., 2023), a total of 40 male SD rats (8 weeks, male; purchased from HFKbio, China) were randomly divided into four groups (n = 10) by simple randomization method, using a computer-generated table: normal (saline); MPS (Methylprednisolone hemisuccinate (MPS) + saline); EVs^No PEMFs^ (MPS+EVs without preconditioning); and EVs^PEMFs^ (MPS+proper EVs^PEMFs^). The model and treatment protocols were established as previously reported. MPS (20 mg/kg per day; TargetMol, China) or saline was injected intramuscularly into rats to induce ONFH during the first 3 days of each week, continuing until the third week (Zhao et al., 2023). Beginning with the first injection of MPS, the EVs (100 μL, 10^10^ particles) were injected into the rats through the tail vein, thrice a week for 3 weeks (Chen et al., 2020; Guo et al., 2016). Five rats were dissected after isoflurane overdose for examination at 2 weeks and the remaining rats were dissected at 6 weeks after the first injection (Chen et al., 2022).

### 2.12 Micro-CT analysis

The femoral heads were scanned using micro-CT (Quantum GX, PerkinElmer, United States), with a voltage of 80 kV and a current of 100 μA. The scanner software was configured to achieve high resolution, utilizing a voxel size of 20 μm and a field of view measuring 10 mm. Three-dimensional (3D) images of the femoral heads were reconstructed and the bone volume/total volume (BV/TV), bone surface/bone volume (BS/BV), bone mineral density (BMD), trabecular number (Tb.N), trabecular thickness (Tb.Th), and trabecular separation (Tb.Sp) of the region of interest was calculated by Caliper Analyze (BIR, Mayo Clinic, United States).

### 2.13 Histological and immunohistochemistry analysis

The specimens for histological evaluation, including hematoxylin and eosin (H&E) and Masson’s trichrome staining, were processed according to earlier protocols (Li G. et al., 2020). Images were captured using a light optical microscope (Ni-E, Nikon, United States). The specimens were decalcified, fixed in formaldehyde, dehydrated, and embedded in paraffin. After dewaxing and antigen retrieval, 10% bovine serum albumin was used to block nonspecific binding for 30 min. The sections were incubated overnight at 4 °C with primary antibodies (seen in Supplementary Table S5) and then with an HRP-conjugated secondary antibody (PV9001, ZSGB Biotechnology, China), counterstained with hematoxylin. All tests were done on at least three sections per specimen and regions of interesting were selected randomly by two independent observers to ensure consistency and representativeness while minimizing sampling bias.

### 2.14 Statistical analysis

The data are expressed as the mean ± standard deviation (SD) based on a minimum of three separate experiments. Statistical analysis was conducted using GraphPad Prism 9 (La Jolla, CA, United States). One-way ANOVA was used to compare group means, with Bonferroni’s *post hoc* analysis for significance between pairs. A *p*-value <0.05 was deemed significant.

## 3 Results

### 3.1 Characterization of BMSCs and PEMFs with different amplitudes preconditioned BMSC-EVs

**FIGURE 1:**
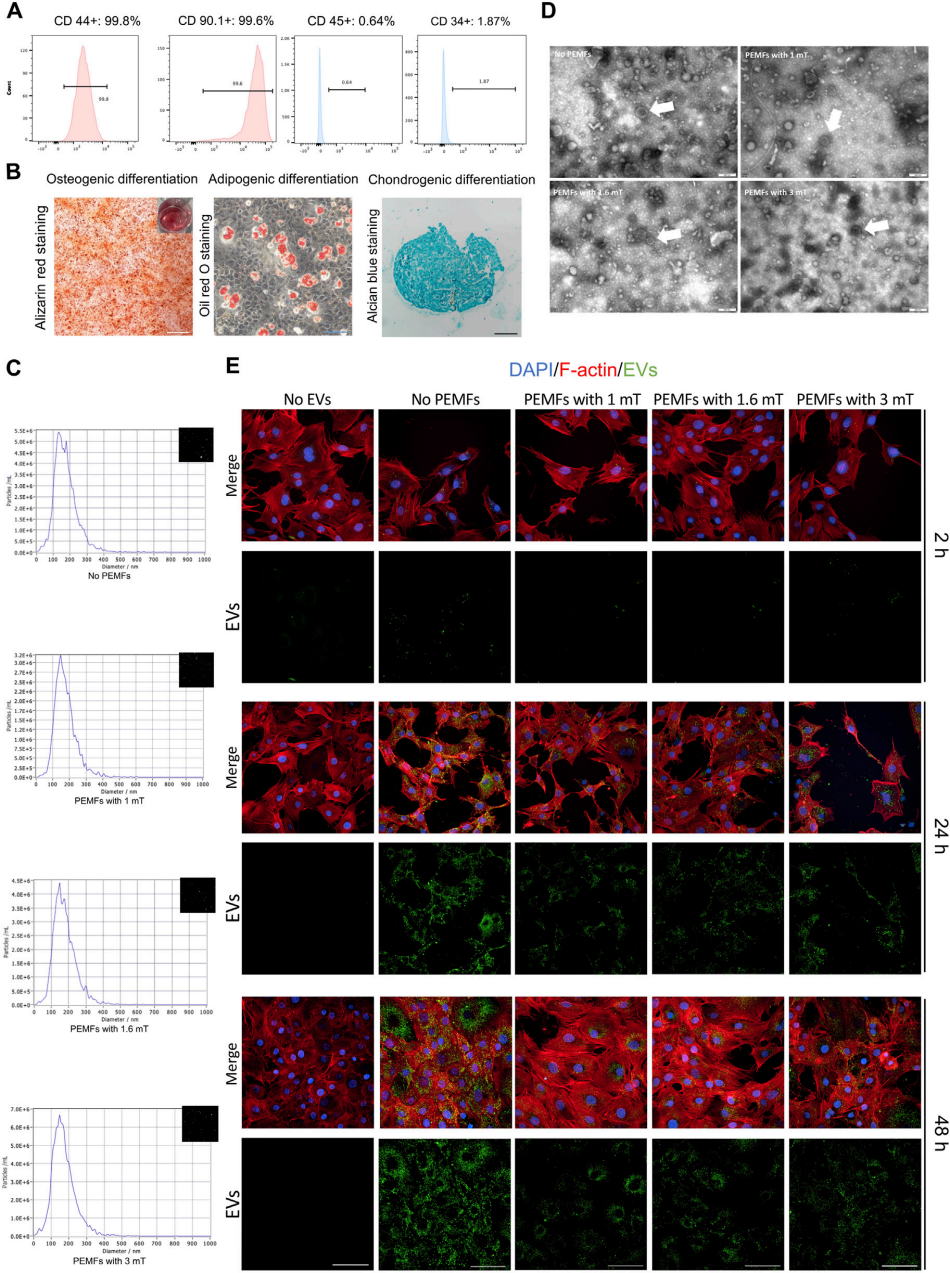
Characterization of rat BMSC-EVs under different amplitudes of PEMFs exposure system and uptake of BMSC-EVs. **(A)** Flow cytometric analysis of the surface markers of BMSCs. **(B)** The osteogenic differentiation, adipogenic differentiation, and chondrogenic differentiation of MSCs. Scale bars: 500 μm (white), 200 μm (black), and 100 μm (blue). **(C)** The particle size distribution of BMSC-EVs with different parameters of PEMFs. **(D)** A representative TEM image of BMSC-EVs from supernatant under different parameters of PEMFs. White arrows: representative images of BMSC-EVs. Scale bars: 200 nm. **(E)** Cellular uptake assay by the confocal system of high-content screening demonstrated uptake of BMSC-EVs by MC3T3-E1 cells after 2, 24, and 48 h (BMSC-EVs: green; MC3T3-E1 cytoskeleton: red; MC3T3-E1 nucleus: blue). Scale bars: 100 μm.

BMSCs expressed CD44 and CD90, while showing no expression of CD34 and CD45 (Figure 1A). They demonstrated the capacity to differentiate into adipocytes, osteoblasts, or chondrocytes upon appropriate induction (Figure 1B). The microscopy view indicated typical shape of BMSCs, and good viability from colonies (Supplementary Figure S4A). The EVs (BMSC-EVs and BMSC-EVs^PEMFs^) were smaller than 200 nm (Figure 1C) with a mean diameter of 97.30 ± 11.34 nm, 95.00 ± 8.26 nm, 103.50 ± 10.20 nm and 99.60 ± 6.78 nm in BMSC-EVs^No PEMFs^, BMSC-EVs^PEMFs (1 mT)^, BMSC-EVs^PEMFs (1.6 mT)^, and BMSC-EVs^PEMFs (3 mT)^ group, respectively. The EVs exhibited a round-shaped morphology (Figure 1D). No obvious difference of particles numbers was detected (*p* > 0.05, Supplementary Figure S4B). Moreover, particles were positive for EVs markers including CD9, CD81, and TSG101, and negative for Calnexin (endoplasmic marker, Supplementary Figure S4C). The result of immunofluorescence manifested that the DIO-labeled EVs could transfer to the perinuclear region of BMSCs after incubation with BMSCs (Figure 1E). The viability of osteoblasts was not reduced when incubated with EVs and most EVs transferred to osteoblasts after 24 h incubation (Supplementary Figure S4D). These results indicated that BMSC-EVs were isolated and incorporated into MC3T3-E1 cells.

### 3.2 PEMFs with 3 mT preconditioned BMSC-EVs enhanced the osteogenesis of MC3T3-E1 cells

**FIGURE 2:**
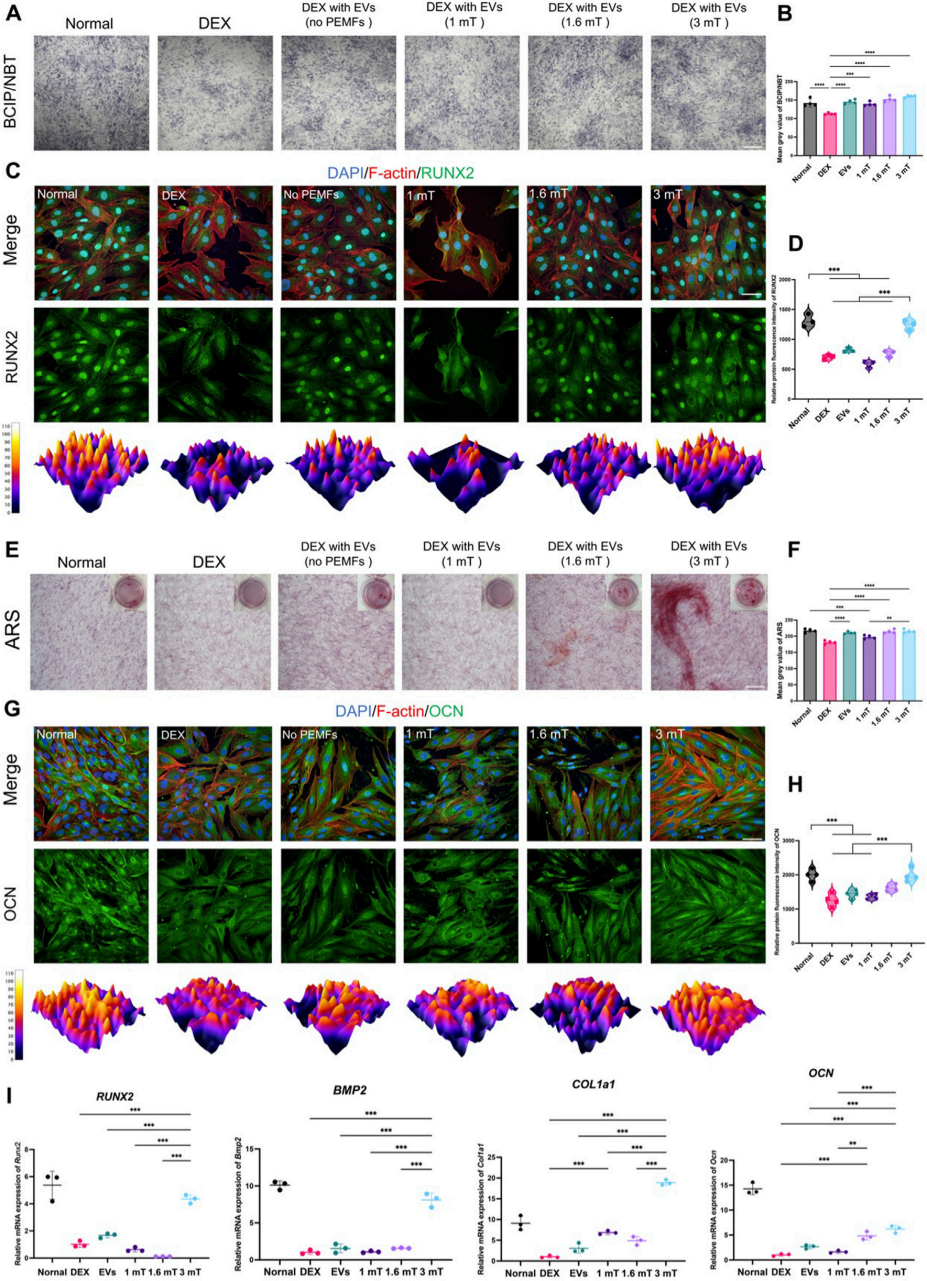
The osteogenesis of MC3T3-E1 cells treated with different BMSC-EVs^PEMFs^. **(A)** Assessment of ALP by BCIP/NBT. Scale bars: 500 μm, and **(B)** Quantification of ALP activity. **(C)** Immunofluorescence images of expression in MC3T3-E1 cells after different treatments for 7 days (RUNX2: green; cytoskeleton: red; nucleus: blue; 3D thermal imaging: reconstruction of fluorescence intensity of RUNX2). Scale bar: 50 μm, and **(D)** Quantitative analysis of immunofluorescence results of RUNX2. **(E)** Assessment of mineralization by alizarin red staining. Scale bar: 500 μm, and **(F)** Quantitative analysis of alizarin red staining. **(G)** Immunofluorescence images of expression in MC3T3-E1 cells after different treatments for 21 days (OCN: green; cytoskeleton: yellow; nucleus: blue; 3D thermal imaging: reconstruction of fluorescence intensity of OCN). Scale bar: 50 μm. **(H)** Quantitative analysis of immunofluorescence results of OCN. **(I)** Expression of Runx2, Bmp2, Ocn and Col1a1 mRNA was measured by RT-qPCR. Bars represent mean and SD. Compared with each group as determined by one-way ANOVA and *post hoc* analysis, where *p < 0.05, **p < 0.001, ***p < 0.0001.

All intervention groups displayed an enhancement of osteogenic activity, with the effect of BMSC-EVs^PEMFs (3 mT)^ being significantly stronger than that of different intensities and BMSC-EVs^No PEMFs^. The activity of ALP was higher in BMSC-EVs^PEMFs (3 mT)^ group (*p* < 0.001, Figures 2A,B). The protein level of RUNX2 after 7-day induce were significantly higher than other groups with DEX (*p* < 0.001, Figures 2C,D). RT-qPCR determined that the expression of *Runx2* and *Bmp2* in BMSC-EVs^PEMFs (3 mT)^ group were significantly higher than that in the DEX, EVs, and other amplitudes groups (*p* < 0.001, Figure 2I). Subsequently, the result of ARS staining after 21-day differentiation demonstrated that the calcium nodules in EVs, BMSC-EVs^PEMFs (1.6 mT)^ and BMSC-EVs^PEMFs (3 mT)^ groups were significantly higher than that in the DEX groups (*p* < 0.001, Figures 2E,F). OCN was high-expressed in BMSC-EVs^PEMFs (3 mT)^ group than EVs and BMSC-EVs^PEMFs (1.6 mT)^ group (*p* < 0.001, Figures 2G,H). Moreover, the expression of *Ocn* and *Col1a1* in the BMSC-EVs^PEMFs (3 mT)^ group was be increased comparing to DEX, EVs, and other amplitude groups (*p* < 0.001, Figure 2i).

### 3.3 Comparison of mRNAs revealed the mechanism of BMSC-EVs^PEMFs (3 mT)^ therapy

**FIGURE 3:**
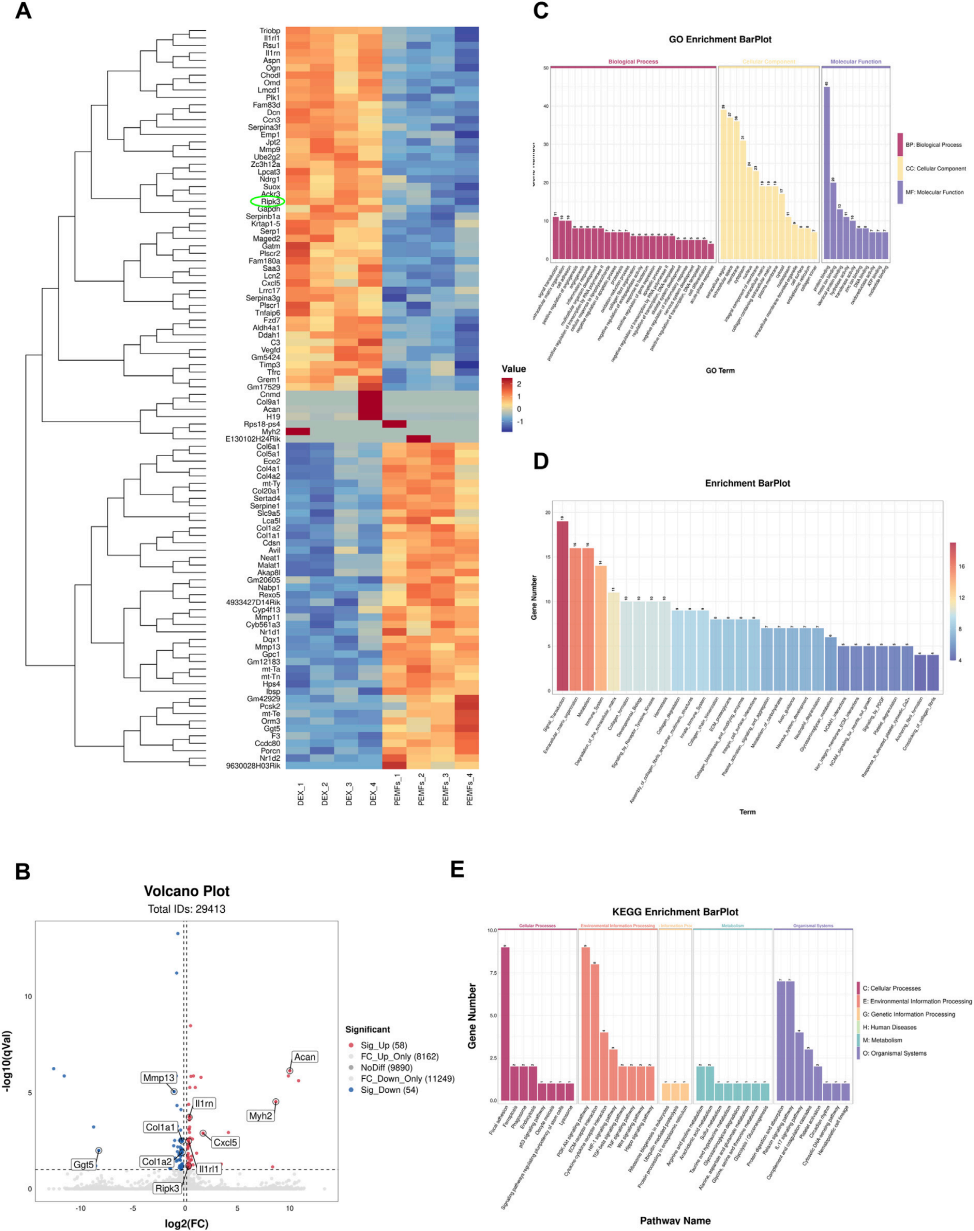
RNA-Seq analysis in MC3T3-E1 treated with BMSC-EVs^PEMFs (3 mT)^ and negative control. **(A)** Heatmap depicting the expression of different expression genes (DEGs) in groups, which was extracted from DEX group (n = 4) and DEX with EVs^PEMFs^ ^(3 mT)^ group (n = 4). The top 100 mRNAs with the highest fold-change were identified, and cell programmed death-related gene is circled. **(B)** Volcanic map of DEGs between groups. Red spots represent upregulated genes and blue spots represent downregulated genes. **(C)** Enrichment plot of GO annotations. The top significant GO enrichments of the target genes of the DEGs are shown. The correlation between mRNA and related biological processes, cellular component, and molecular function was measured using the negative log_10_ of the q-value. GO, Gene Ontology. **(D)** The Reactome enrichment analysis scatter plot. **(E)** KEGG enrichment for groups. The top 8 most relevant KEGG pathways of the DEGs. KEGG, Kyoto Encyclopedia of Genes and Genomes.

Based on mentioned results, BMSC-EVs^PEMFs (3 mT)^ and the DEX group were conducted for RNA sequencing. The heatmap demonstrated the top 100 genes according to relative expression (Figure 3A). The volcano plot analysis indicated 58 DEGs were upregulated and 54 DEGs were downregulated in the DEX compared with the BMSC-EVs^PEMFs (3 mT)^ (Figure 3B). There results confirmed that the programmed cell death-related gene, *Ripk3*, was significantly reduced in BMSC-EVs^PEMFs (3 mT)^ group. Furthermore, GO database analysis indicated mechanism involved in bone development and cell death (Figure 3C). Reactome pathway database analysis determined several pathways were involved, such as ECM synthesis, metabolism, immune system, collagen synthesis (Figure 3D). The relevant enriched KEGG pathways analysis in Figure 3E indicated that the regulated genes clustered in cell adhesion and communication, immune-inflammatory regulation, and bone mentalism (such as TGF-beta, Wnt, and Hippo signaling pathways).

### 3.4 BMSC-EVs^PEMFs^ reduced the level of necroptosis via inhibition of RIPK1–RIPK3–MLKL signaling

**FIGURE 4:**
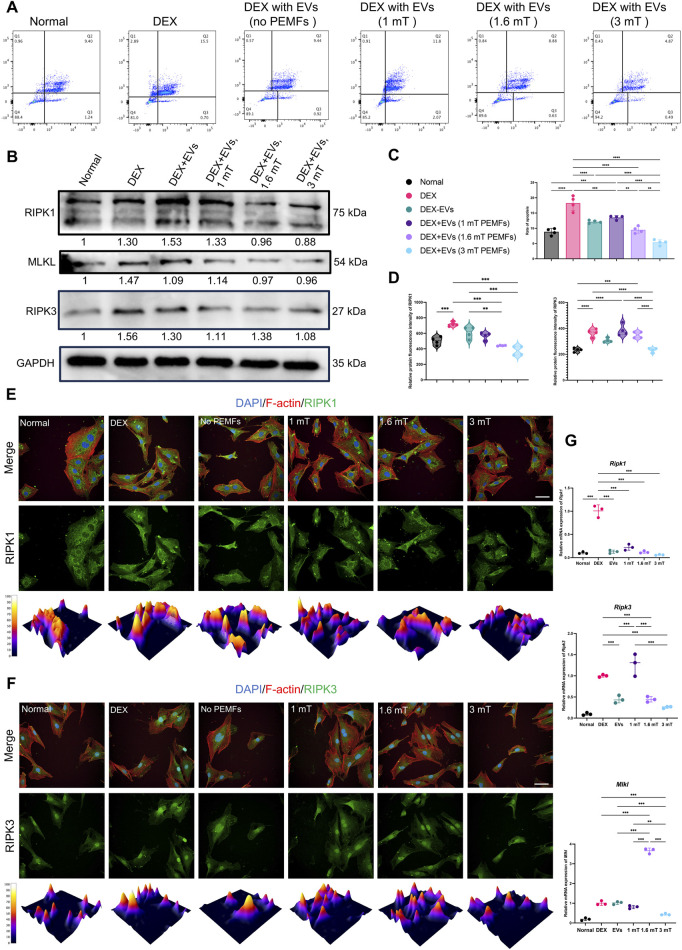
Effect of the different BMSC-EV^PEMFs^ on necroptosis of MC3T3-E1 cells and activation of the necroptosis pathway. **(A)** The programmed cell death of MC3T3-E1 cells estimated with Annexin V/PI staining and reflected in Q2, and **(C)** the rate of programmed cell death of MC3T3-E1 cells estimated with Annexin V/PI staining. **(B)** Representative western blots of RIPK1, RIPK3, and MLKL proteins in MC3T3-E1 cells treated with the BMSCs-EVs under different intensities of PEMFs. GAPDH was used as internal loading control (gel was cut at 70 and 40 kDa and the original images are presented in Supplementary Figures S5–S9). **(D)** Relative protein immunofluorescence intensity of RIPK1 and RIPK3. **(E,F)** Immunofluorescence images of RIPK1 and RIPK3 expression in MC3T3-E1 cells after different treatments. (RIPK1/RIPK3: green; cytoskeleton: red; nucleus: blue; 3D thermal imaging: reconstruction of fluorescence intensity of RIPK1). Scale bar: 50 μm. **(G)** Expression of *Ripk1*, *Ripk3*, and *Mlkl* mRNA was measured by RT-qPCR. Bars represent mean and SD. The significant difference was analyzed by one-way ANOVA and *post hoc* analysis, where *p < 0.05, **p < 0.001, ***p < 0.0001.

Annexin V/PI staining indicated that higher percentage of cell with programmed cell death in the DEX group than that other groups and the BMSC-EVs^PEMFs (3 mT)^ significantly ameliorated the ratio of dying cells (*p* < 0.001, Figures 4A,C). Western blot in Figure 4B (full-length gels are presented in Supplementary Figures S5–S9) determined a decrease in RIPK1, RIPK3, and mixed lineage kinase domain-like (MLKL). Immunofluorescence analysis in Figures 4E,F and RT-qPCR (Figure 4G) indicated, and RT-qPCR indicated BMSC-EVs^PEMFs (3 mT)^ abolished the increase of RIPK3 and MLKL (*p* < 0.001) than DEX group, suggesting a therapeutic effect on inhibiting necroptosis.

### 3.5 BMSC-EVs^PEMFs (3 mT)^ improved bone morphology of the femoral head

**FIGURE 5:**
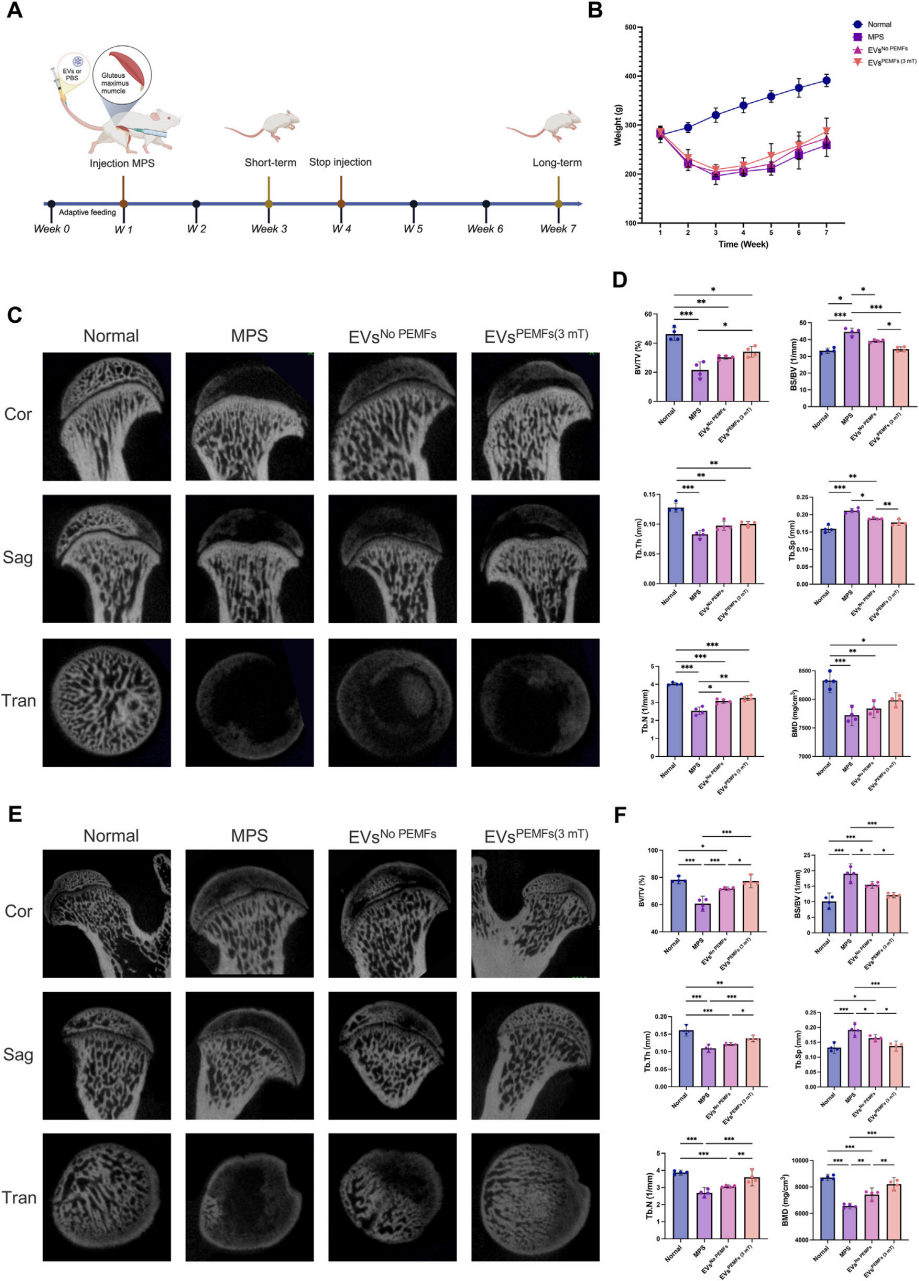
Effect of the BMSC-EVs assessed by micro-CT images of the femoral heads. **(A)** Schematic display of methods and time nodes for injection *in vivo*. **(B)** The weight record of each group (week 1–3: n = 10; week 4–7: n = 5). **(C)** Micro-CT reconstructed images of femoral heads, including a coronal 2D image, a sagittal 2D image, a transverse section, and a 3D reconstruction of ROI at 2 weeks after the first injection (short-term period), and **(D)** quantitative analysis. **(E)** Micro-CT reconstructed images of femoral heads, including a coronal 2D image, a sagittal 2D image, a transverse section, and a 3D reconstruction of ROI at 6 weeks after the first injection (long-term period) and **(F)** quantitative analysis of the ROI. n = 5 per group. Bars represent mean and SD. The significant difference was analyzed by one-way ANOVA and *post hoc* analysis, where *p < 0.05, **p < 0.001, ***p < 0.0001.

ONFH rat models were used to investigate if BMSC-EVs^PEMFs (3 mT)^ could effectively prevent the development of ONFH *in vivo* (Figure 5A). The normal, MPS, EVs^No PEMFs^, and EVs^PEMFs (3 mT)^ group were established for comparison, and the weights of each group were recorded (Figure 5B). Images of the coronal, sagittal, and transverse planes of micro-CT analyzing short-term effect srevealed that the MPS group suffered significant bone mineral loss, decreased bone density, and the presence of osteonecrosis-like structures below the epiphyseal line compared to the normal group. However, these adverse effects were somewhat improved in the EVs^No PEMFs^ and EVs^PEMFs (3 mT)^ groups, although the results still fell short of expectations (Figure 5C). The BS/BV of the region of interest was significantly increased in EVs^PEMFs (3 mT)^ group compared to EVs^No PEMFs^ group (*p* < 0.01, Figure 5D). In the long-term observations, images of the planes revealed that the EVs^PEMFs (3 mT)^ group displayed a compact and evenly distributed trabecular bone structure. This suggests that the EVs^PEMFs (3 mT)^ eliminated the negative effect to the femoral head caused by MPS. However, the EVs^No PEMFs^ still did not meet expectations (Figure 5E). The BV/TV, BS/BV, Tb.Th, Tb.N and BMD were significantly increased in EVs^PEMFs (3 mT)^ group compared to EVs^No PEMFs^ group, while Tb.Sp was decreased (*p* < 0.05, Figure 5F).

### 3.6 BMSC-EVs^PEMFs (3 mT)^ inhibited necroptosis and promoted bone formation of femoral head

**FIGURE 6:**
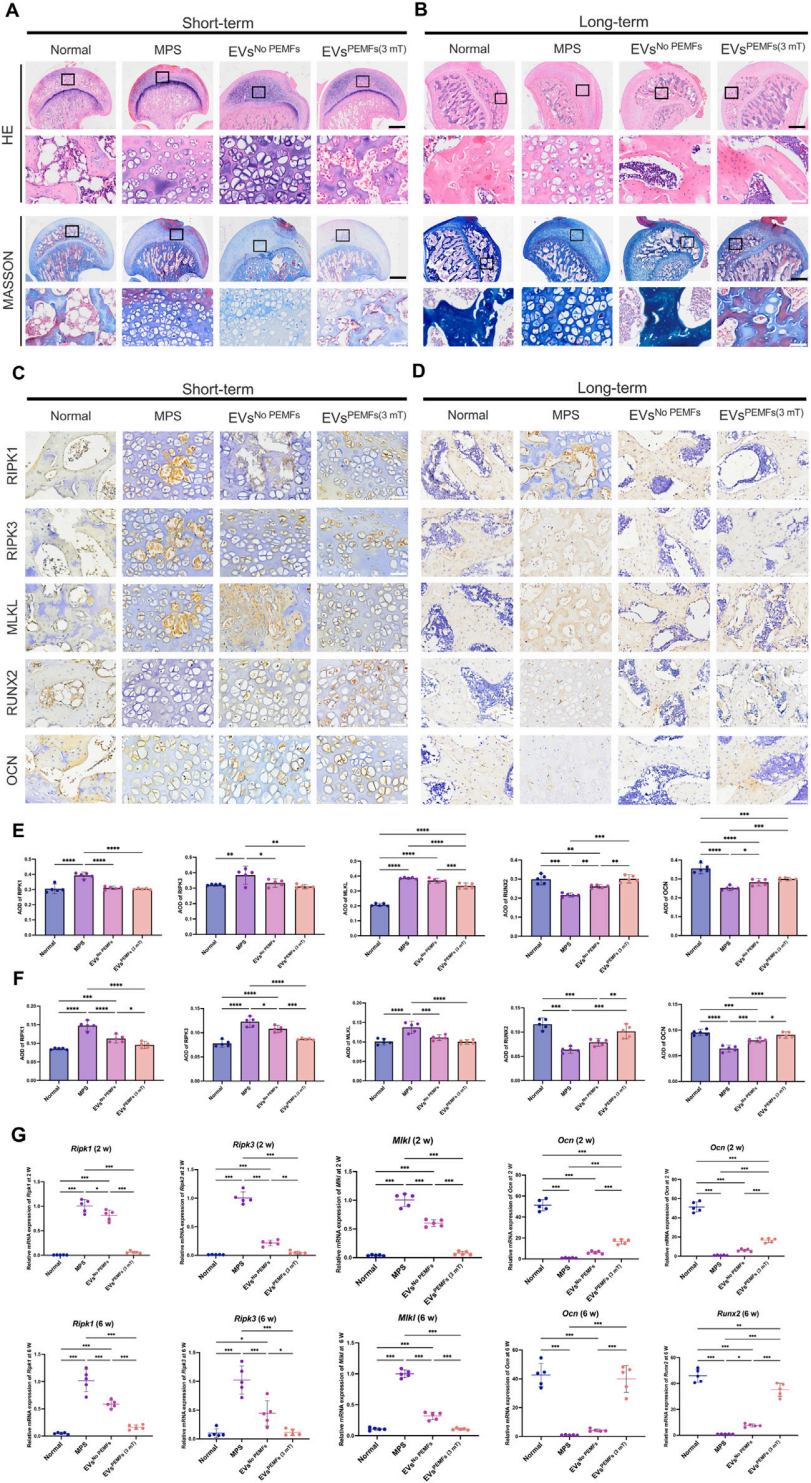
The anti-necroptosis and osteogenesis-promoting effects of EVs on the rat model of ONFH. **(A)** Representative H&E and Masson staining images of femoral heads in rats receiving different treatments at week 2 and **(B)** Week 6 after the first injection. Scale bars: 750 μm (black) and 100 μm (white). **(C)** Representative immunohistochemical staining of RIPK1, RIPK3, MLKL, OCN, and ALP in samples from different groups at week 2 and **(D)** Week 6 after the first injection. Scale bars: 100 μm. Black arrow: RIPK1, RIPK3, MLKL, OCN, or ALP positive cells. **(E)** Quantitative analysis of the level of RIPK1, RIPK3, MLKL, OCN, and ALP in femoral heads from each group at week 2 and **(F)** Week 6 after the first injection. The black arrow indicates the positive cells, n = 5 per group. **(G)** Expression of *Ripk1*, *Ripk3*, *Mlkl*, *Ocn*, and *Alp* mRNA at week 2 and week 6 after the first injection was measured by RT-qPCR (n = 5). Bars represent mean and SD. The significant difference was analyzed by one-way ANOVA and *post hoc* analysis, where *p < 0.05, **p < 0.001, ***p < 0.0001.

H&E staining Figure 6A revealed significant formation of empty bone lacunae, localized disruption of bone trabeculae characterized by a sparse and disorganized structure, as well as the invasion of adipose tissue into the marrow cavity in the short-term samples of the femoral head from the MPS and EVs^No PEMFs^ groups. Most areas of the femoral head in EVs^PEMFs (3 mT)^ group also presented the above changes. Masson staining indicated some new bone trabeculae transitioning from blue to red in EVs^PEMFs (3 mT)^ group. For dissection at long-term shown in Figure 6B, HE staining revealed that the trabecular bone was still occupied by an abundance of cells resembling adipocytes, along with deteriorating cells exhibiting condensed nuclei and encircled by a lucid cytoplasmic area in MPS group, and the trabecular bone became sparse and thin in EVs^No PEMFs^ group. In contrast, rats after the treatment of EVs^PEMFs (3 mT)^ showed only slight osteonecrosis of the trabecular bone, as well as fewer empty lacunae and adipose cells. Short-term immunohistochemistry analysis revealed lower expression of MLKL, and higher expression of RUNX2 in EVs^PEMFs (3 mT)^ group (*p* < 0.05, Figures 6C,E). For long-term, lower expression of RIPK1 and RIPK3, and higher expression of RUNX2 and OCN in EVs^PEMFs (3 mT)^ group than others (*p* < 0.05, Figures 6D,F). Furthermore, more mRNA of *Ripk1*, *Ripk3*, and *Mlkl* were expressed in MPS group (*p* < 0.05), and more mRNA of *Ocn* and *Runx2* were expressed in normal group than others (*p* < 0.05) (Figure 6G).

**FIGURE 7:**
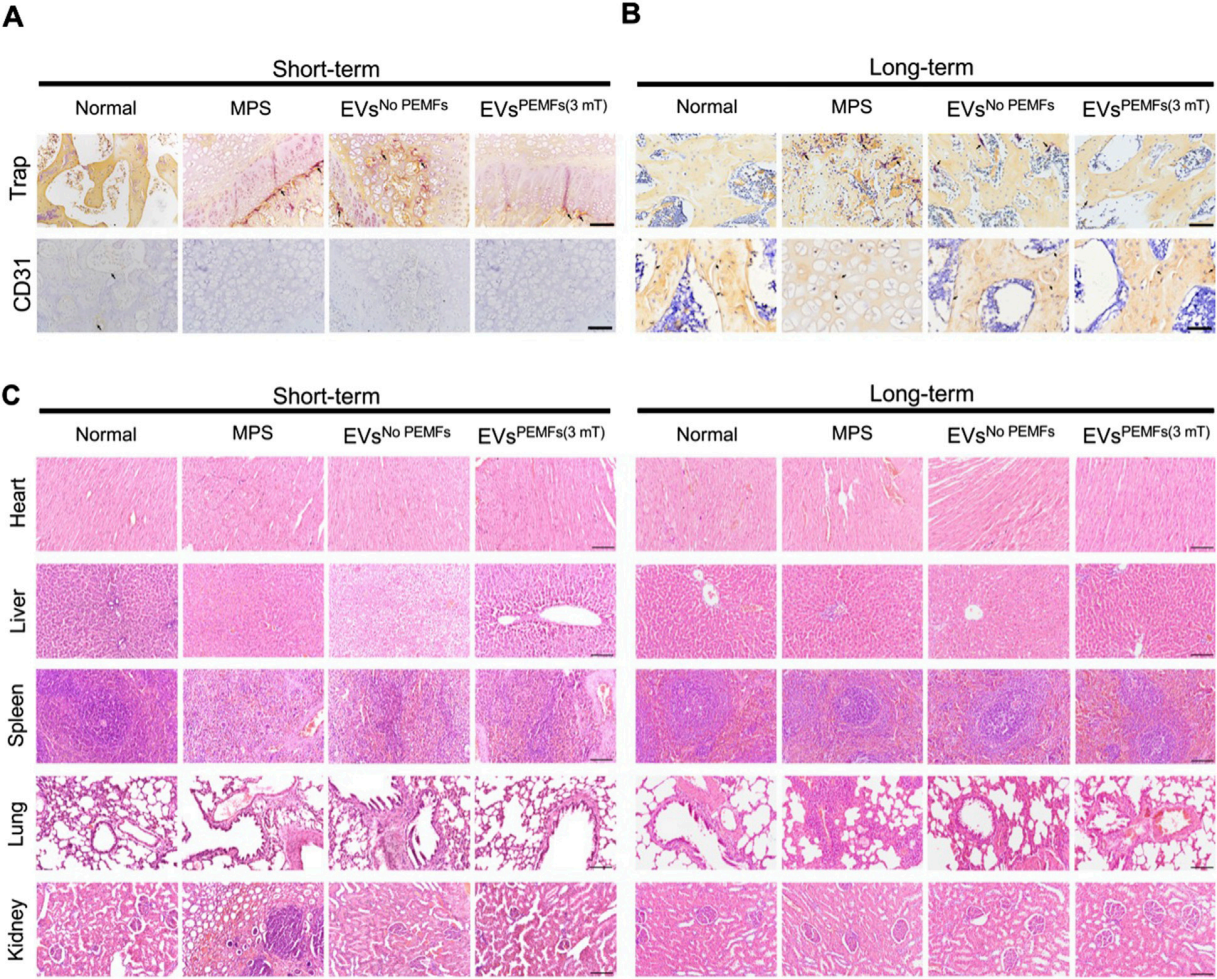
The effects of EVs on anti-osteoclasts, angiogenesis and important organs. **(A)** Representative immunohistochemical staining of Trap and CD31 in samples from different groups at week 2 and **(B)** week 6 after injection. Scale bars: 100 μm. Black arrow: Trap/CD31-positive cells. **(C)** H&E staining of major organs (heart, liver, spleen, lung, and kidneys, respectively) at week 2 and week 6. Scale bars: 100 μm.

Immunohistochemical staining for CD31 and TRAP was conducted to investigate the distribution of blood vessels and osteoclasts within the femoral heads across various groups. The normal, EVs^No PEMFs^, and EVs^PEMFs (3 mT)^ groups had higher expression of CD31, and the MPS group displayed the lowest levels (Figure 7A). TRAP staining indicated that the osteoclasts in normal and EVs^PEMFs^ group were inactive than others (Figure 7B). The results confirmed that the MPS injections in rats led to a significant reduction in blood vessel formation and increased activity of osteoclasts in the femoral head. In contrast, BMSC-EVs^PEMFs (3 mT)^ effectively mitigated blood vessel deficiency and osteoclast activity, avoiding the deterioration of ONFH. Additionally, HE staining of vital organs showed no significant biotoxicity from BMSC-EVs and BMSC-EVs^PEMFs (3 mT)^ to, although nephrocalcinosis was observed in the MPS group (Figure 7C).

## 4 Discussion

In this study, we used the easily available BMSCs and manufactured PEMFs to engineer the EVs secreted by BMSCs. The optimal field amplitude might be 3 mT, and BMSC-EVs^PEMFs (3 mT)^ showed more positive potential for anti-necroptosis and osteogenesis. Additionally, we provided evidence that administering BMSC-EVs intravenously under PEMFs at a field amplitude of 3 mT during the initial glucocorticoid exposure stages significantly inhibited trabecular bone cell necroptosis, restored compromised angiogenesis, and averted trabecular bone deterioration in the femoral heads of rats. Meanwhile, we identified the involvement of RIPK1, RIPK3, and MLKL proteins in the anti-necroptotic effects of BMSC-EVs^PEMFs (3 mT)^ in cultured osteoblast precursors exposed to DEX and in ONFH rats induced by MPS. Our study indicates the promising prospect of BMSC-EVs as a nanoparticle-based tool for safeguarding against GC-induced ONFH. Furthermore, the EVs derived from BMSCs cultured under PEMFs at a field amplitude of 3 mT enhanced the effects. Targeting molecules such as RIPK1, RIPK3, and MLKL could further enhance their protective functions, opening exciting avenues for research and potential treatments.

Previous studies more focused on the role of apoptosis in ONFH. Yang et al. (2021) and Tao et al. (2017) both focused on apoptosis and illustrated the mechanism by which apoptosis attenuates ONFH and that exosomes from platelet-rich plasma prevent apoptosis. Nonetheless, a recent study has demonstrated that necroptosis plays an important role in the development of ONFH (Fan et al., 2022). For musculoskeletal diseases, Yuan and their colleague have reported that bone marrow necroptosis can lead to myelodysplasia which was mediated by the over-expression of *Ripk1* (Yuan et al., 2019). RIPK1 is an important molecular activated by the death receptor and leads to the activity-dependent formation of a RIPK1-RIPK3-MLKL complex (also known as complex IIb). In addition to its role in necroptosis, Lawlor and colleagues reported that RIPK3 is associated with NLRP3 inflammasome. On the one hand, RIPK3 with active caspase-8 promoted apoptosis and NLRP3–caspase-1 activation. On the other hand, in the absence of caspase-8, RIPK3 kinase activity and MLKL are essential for Toll-like receptor-induced NLRP3 activation (Lawlor et al., 2024). In our study, differences in Ripk3 expression were detected after the treatment of BMSC-EVs^PEMFs (3 mT)^. For ONFH, Dai et al. and their colleagues demonstrated necroptosis of osteoblasts mediated by RIPK1, RIPK3, and MLKL with relative resistance to apoptosis (Dai et al., 2020). Hence, we further investigated the changes in RIPK3 and MLKL, and focused on the Ripk1–Ripk3–Mlkl mediated necroptosis in ONFH.

EVs transfer into the extracellular space through the plasma membrane while protecting their contents by the lipid structure, hence inherently benefit from immune tolerance (Thakur et al., 2022). Previous studies have reported the therapeutic effects of EVs derived from CD34^+^ stem cells (Zuo et al., 2019), adipose-derived stem cells (Nan et al., 2021), BMSCs (Li L. et al., 2020), and synovial-derived MSCs (Guo et al., 2016) on preventing ONFH via increased proliferation and osteogenic differentiation of BMSCs. Our study focused on the function of osteoblasts, which are major mediator for bone formation (Dirckx et al., 2019). Additionally, an increasing number of researchers have focused on methods for enhancing the function of EVs. Chen et al. engineered EVs with hydrogel to improve bone repair capabilities (Chen et al., 2023). Besides tissue engineering, changing the condition of original cells also mediates the contents in EVs. Tian and colleagues harvested EVs from dental pulp stem cells subjected to hypoxic preconditioning. Their findings revealed that these hypoxia-derived EVs can promote the generation of M2 macrophages while concurrently suppressing osteoclastogenesis (Tian et al., 2023).

PEMFs seem to be convenient physical therapy for engineering EVs and enhancing the therapeutic role of EVs. PEMFs may activate the BMP2 pathway via notable Ca^2+^ oscillations with robust Ca^2+^ spikes (Yan et al., 2022; So et al., 2000). However, the parameters of PEMFs are always controversial and different to be consistent for targeting varied cells. Wong and their colleague indicated that a single 10-min exposure of donor myoblast cultures to 15 or 50 Hz with 1.5 mT amplitude PEMFs can stimulate EVs release and the conditioned medium with EVs demonstrate similar growth and survival potentials when compared to traditional fetal bovine serum (Wong et al., 2022). Our previous study investigated the effect of MSC-EVs under PEMFs exposure at 1 mT amplitude with different frequencies of 15, 45, and 75 Hz on reducing IL-1β-induced chondrocyte inflammation. The results demonstrated that PEMFs with 75 Hz obviously regulated the biofunction of MSC-EVs (Xu et al., 2022). Parate and their colleague investigated the effect of PEMFs at a frequency of 15 Hz with 1–4 mT amplitude on BMSC chondrogenic differentiation, and the RT-qPCR and secretome analysis indicated that 3 mT was the best amplitude for two-dimensional culture (Parate et al., 2020). In this study, we chose the PEMFs at a frequency of 75 Hz with 1–3 mT amplitude, and we found 3 mT amplitude might be the optimal intensity for osteogenic differentiation of osteoblasts. The conclusion was partly similar to Parate and their colleague, but the PEMFs exposure frequency was different, which might be caused by the character of targeted cells. Our results evidenced that BMSC-EVs^PEMFs (3 mT)^ effectively alleviate the development of ONFH by promoting osteogenesis and inhibiting necroptosis of osteoblasts via Ripk1–Ripk3–Mlkl signaling, which still warrants future investigation.

Although we demonstrated the effect of BMSC-EVs^PEMFs (3 mT)^, several limitations were shown in this study, Initially, we established MPS-induced ONFH models in SD rats through intramuscular injections of MPS for three consecutive days each week over a 3-week period. We observed characteristic pathological features of ONFH, including notable and consistent bone lesions in the femoral heads without femoral head collapse. We subsequently administered the same types of therapies via tail vein injection. This type of injection can only be used for the early stage of the disease or for preventing ONFH, since the damage to blood vessels of the femoral head worsens with the progression of the disease. Moreover, we only tested CD31 while more angiogenic and vasculogenic markers and functional assays should be conducted for a comprehensive understanding of vascular responses in osteonecrosis. It remains unclear whether BMSC-EVs^PEMFs (3 mT)^ could effectively attenuate glucocorticoid-induced ONFH in advanced stages. Finally, further studies are needed to comprehensively understand the mechanisms of PEMFs and the changes in EVs derived from cells under PEMFs exposure.

## 5 Conclusion

To avert the onset and slow the progression of ONFH, we implemented the PEMFs exposure system as a convenient physical therapy to engineer the EVs secreted by BMSCs. A novel BMSC-EVs^PEMFs^ was successfully collected with cell-entrance abilities. Cell experiments demonstrated that BMSC-EVs with 3 mT amplitude PEMFs could markedly increase the capacity of osteogenesis and inhibit necroptosis of glucocorticoids-induced osteoblasts via the Ripk1–Ripk3–Mlkl signaling. In ONFH rat models, we further confirmed the outstanding therapeutic efficacy of EVs derived from BMSCs under PEMFs exposure at 3 mT amplitude in the prevention of ONFH. Therefore, the PEMFs exposure system shows great promise as a physical agent. The notable preventive effects of EVs derived from BMSCs under PEMFs exposure at 3 mT on ONFH offer exciting new insights and innovative ideas for treating ONFH and other conditions related to osteogenesis and necroptotic disorders.

## Data Availability

The original contributions presented in the study are publicly available. This data can be found here: https://www.ncbi.nlm.nih.gov/sra/PRJNA1115973.

## Abbreviations

ONFH, Osteonecrosis of the femoral head; BMSC-EVs, Extracellular vesicles derived from bone marrow mesenchymal stromal cells; EVs, Extracellular vesicles; PEMFs, Pulsed electromagnetic fields; BMSCs, Bone marrow mesenchymal stromal cells; BMSC-EVs^PEMFs^, PEMFs preconditioned BMSCs-EVs; mT, milli Tesla; RIPK, Receptor-interacting protein kinase; DEX, Dexamethasone; MLKL, Mixed lineage kinase domain-like; ARS, Alizarin red staining; TEM, Transmission electron microscopy; NTA, Nanoparticle trafficking analysis; ALP, Alkaline phosphatase; HRP, Horseradish peroxidase; RT-qPCR, Reverse transcription-quantitative polymerase chain reaction; SD, Sprague-Dawley; MPS, Methylprednisolone hemisuccinate; BV/TV, Bone volume/total volume; BS/BV, Bone surface/bone volume; BMD, Bone mineral density; Tb.N, Trabecular number; Tb.Th, Trabecular thickness; Tb.Sp, Trabecular separation; H&E, Hematoxylin and eosin; SRA, Short Read Archive; DEGs, Differentially expressed genes; KEGG, Kyoto Encyclopedia of genes and genomes.
