# Supplementary material for: Pulsed electromagnetic fields preconditioned extracellular vesicles derived from mesenchymal stromal cells prevents necroptosis of osteoblasts in osteonecrosis of the femoral head rats
Source: Front Bioeng Biotechnol. 2025 Oct 9;13:1655579. doi: 10.3389/fbioe.2025.1655579 (PMC12547990; doi:10.3389/fbioe.2025.1655579)
Supplement: Supplementary file 1 [file DataSheet1.pdf]

Supplementary Materials

Pulsed electromagnetic fields preconditioned extracellular vesicles derived from mesenchymal stromal cells prevents necroptosis of osteoblasts in osteonecrosis of the femoral head rats

Fig. S1

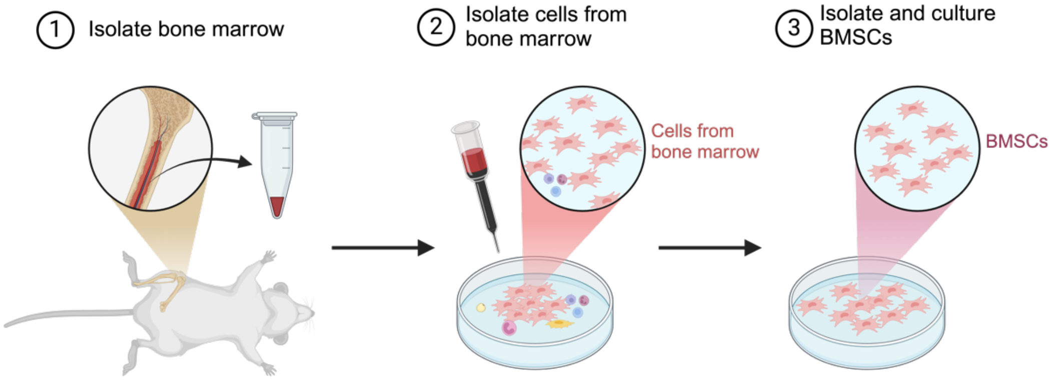

Supplementary Figure S1. The rat BMSC isolation procedures. We flushed bone marrow from the superior alveolar ridge of the cut femur and tibia with α-MEM (Gibco, USA) containing 10% (v/v) fetal bovine serum (FBS; PAN, Germany), then collected.

Fig. S2

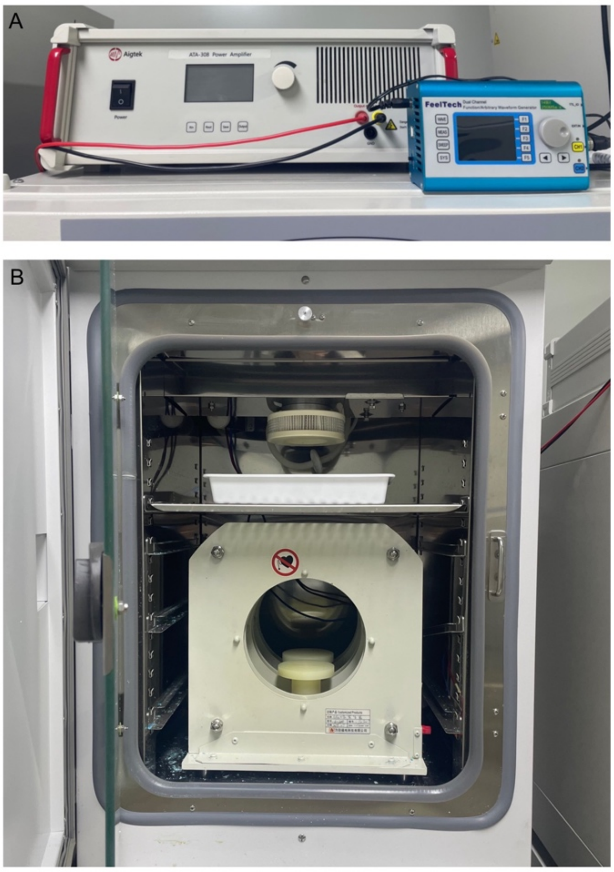

Supplementary Figure S2. The overlook of the PEMFs exposure system. (A) The pulse generator and stepper motor driver. (B) Helmholtz coil in the incubator (open door) and can contain up to eight 15 cm dishes.

Fig. S3

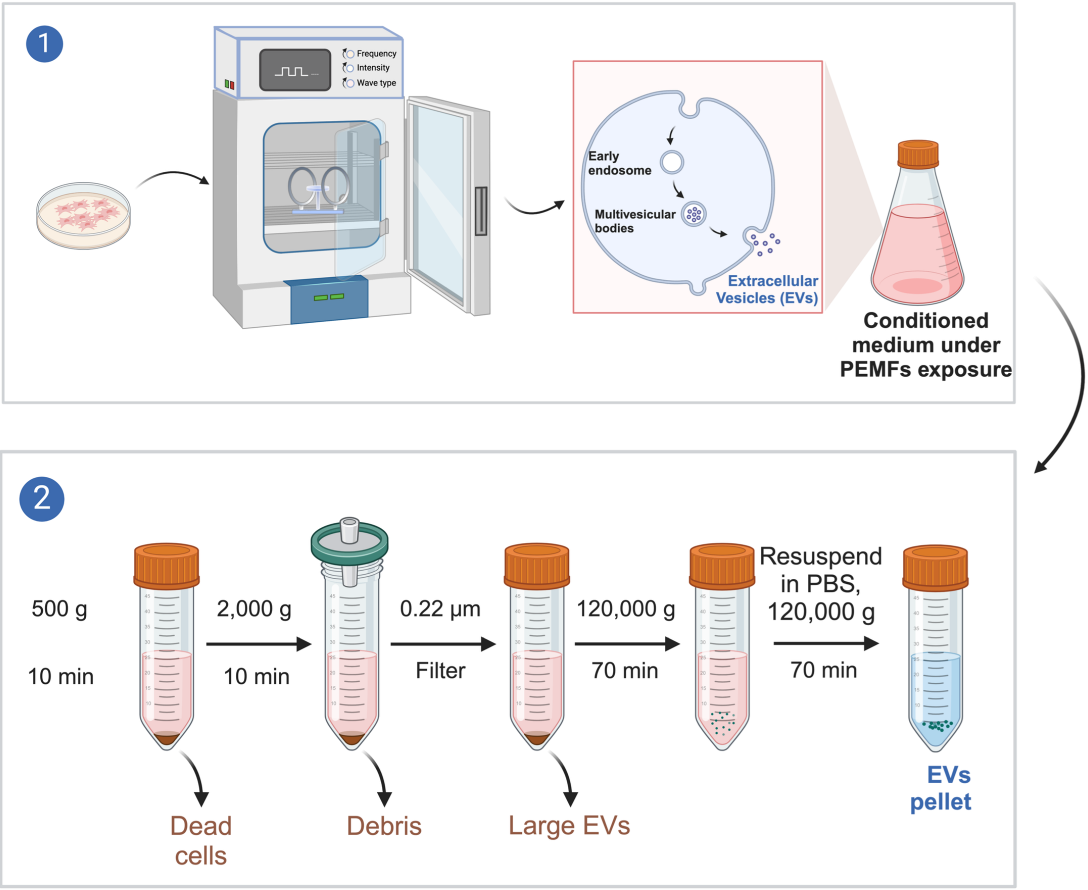

Supplementary Figure S3. The diagram of BMSCs cultured under PEMFs with different amplitudes and isolation procedures of secreted EVs.

Supplementary Table 1. Details of PEMFs device with approval

Test Item	Standard Requirements	Test Results
I. Calculation Accuracy	1. Simulated sinusoidal magnetic field: within the working area, magnetic flux density uniformity ≤ ±0.1%.<br>2. Simulated sinusoidal magnetic field: 0–60 V input voltage, output current 0–10 A. Voltage resolution 0.1 V, current resolution 0.01 A.	1. Sudden simulated magnetic field in the working area: uniformity 0.05%.<br>2. Simulated sinusoidal magnetic field with 0–60 V input voltage: output current 0–10 A.
II. Analog Regulation	1. Magnetic field can be adjusted; field strength varies linearly with input voltage.<br>2. Magnetic field uniformity remains within allowable deviation after adjustment.	During regulation of the magnetic field, uniformity deviation was 0.05%.
III. Magnetic Field Uniformity Test	1. Within the working area, under static magnetic field, uniformity deviation ≤ ±0.3%.<br>2. Within the working area, under dynamic magnetic field, uniformity deviation ≤ 1%.	1. Under static magnetic field in working area: uniformity deviation 0.23%.<br>2. Under dynamic magnetic field in working area: uniformity deviation 0.23%.
IV. Stability Test	1. System should operate continuously without errors during long-term testing.<br>2. Power switch should operate normally during repeated switching.<br>3. Continuous operation should not trigger alarms.<br>4. Continuous operation should maintain stable output.<br>5. During normal voltage fluctuations, the system should remain stable.<br>6. During sudden shutdown, the system should resume properly.<br>7. During abnormal conditions, system should provide protection.	1. Operated continuously for 1.5 h without errors.<br>2. Successfully performed over 4 h of repeated on/off switching.<br>3. No alarms triggered during operation.<br>4. Stable operation maintained, output stability 99.5%.<br>5. Stable under normal voltage fluctuation.<br>6. Recovered normal operation after sudden shutdown.<br>7. Provided protective shutdown under abnormal conditions.
V. Electrical Performance Test	1. Under non-magnetic load, system should deliver normal voltage; voltage accuracy deviation ≤ ±1%.<br>2. Under non-magnetic load, system should deliver normal current; current accuracy deviation ≤ ±1%.<br>3. Under magnetic load, system should deliver normal voltage; voltage accuracy deviation ≤ ±1%.<br>4. Under magnetic load, system should deliver normal current; current accuracy deviation ≤ ±1%.	1. Under non-magnetic load: system voltage deviation 0.75%.<br>2. Under non-magnetic load: system current deviation 0.78%.<br>3. Under magnetic load: system voltage deviation 0.68%.<br>4. Under magnetic load: system current deviation 0.94%.

Supplementary Table 2. Western blot antibody information

Antibody	Cat No	Company	Country	Concentration
CD9	ER80402	Huabio	China	1:1000
CD81	ET1611-87	Huabio	China	1:1000
TSG101	ET1701-59	Huabio	China	1:1000
Calnexin	ER1803-42	Huabio	China	1:1000
RIPK1	AF7896	Beyotime	China	1:1000
RIPK3	AF7893	Beyotime	China	1:1000
MLKL	AF5231	Beyotime	China	1:1000
GAPDH	AB0037	Abways	China	1:1000
HRP-conjugated goat anti-rabbit secondary antibody	PD302	Oriscience	China	1:10000

Abbreviations: CD9, Cluster of Differentiation 9 protein; CD81, Cluster of Differentiation 81 protein; TSG101, tumor susceptibility gene 101 protein; RIPK1, Receptor-interacting serine/threonine-protein kinase 1; RIPK3, Receptor-interacting serine/threonine-protein kinase 3; MLKL, Mixed lineage kinase domain-like protein; GAPDH, Glyceraldehyde-3-phosphate dehydrogenase.

Supplementary Table 3. Primer sequences for RT-qPCR of cells

Genes	Primers	Primer sequence (5'-3')
Col1a1	Forward	GCGAAGGCAACAGTCGCT
	Reverse	CTTGGTGGTTTTGTATTCGATGAC
Runx2	Forward	GGTACTTCGTCAGCATCCTATCAG
	Reverse	GCTTCCGTCAGCGTCAACAC
Bmp2	Forward	GGAAAAGGACATCCGCTCCA
	Reverse	GCCACGATCCAGTCATTCCA
Ocn	Forward	GCAGCTTGGTGCACACCTAG
	Reverse	GGAGCTGCTGTGACATCCAT
Ripk1	Forward	AGTCCTTAGAGGAGGACCAGC
	Reverse	CTCCAGCAGGTCACTGGATG
Ripk3	Forward	GCCTTCCTCTCAGTCCACAC
	Reverse	CTCACCAGAGGAACCGCATA
Mlkl	Forward	GCGTTGGCCCAAATTTGACT
	Reverse	GGTCTTCTGCCTCGTTGACA
Gapdh	Forward	TGCACCACCAACTGCTTAG
	Reverse	GGATGCAGGGATGATGTTC

Abbreviations: RT-qPCR, reverse transcription quantitative polymerase chain reaction; Col1a1, Collagen type I Alpha 1; Runx2, Runt related transcription factor 2; Bmp2, Bone morphogenetic protein 2; Ocn, Osteocalcin; Gapdh, Glyceraldehyde-3-phosphate dehydrogenase; Ripk1, Receptor-interacting serine/threonine-protein kinase 1; Ripk3, Receptor-interacting serine/threonine-protein kinase 3; Mlkl, Mixed lineage kinase domain-like protein.

Supplementary Table 4. Primer sequences for RT-qPCR of femoral head tissue

Genes	Primers	Primer sequence (5'-3')
Col1a1	Forward	TTTCCCCCAACCCTGGAAAC
	Reverse	CAGTGGGCAGAAAGGGACTT
Runx2	Forward	GCGGTGCAAACTTTCTCCAG
	Reverse	TGAACCTGGCCACTTGGTTT
Bmp2	Forward	TGCTTCTTAGACGGACTGCG
	Reverse	GGGGAAGCAGCAACACTAGA
Ocn	Forward	ATTGTGACGAGCTAGCGGAC
	Reverse	AGGGCAACACATGCCCTAAA
Ripk1	Forward	CTTAGACGCGTAGGAGCGGC
	Reverse	GACGGAGCTAGGTGCTGAAG
Ripk3	Forward	GAGACCCAAGCTGAGCCTAA
	Reverse	ACGTCTACAACGTCTACGGC
Mlkl	Forward	GAAAAACCGGGCTGAGTCCT
	Reverse	TTATCCATACCCCGAGTTCCAG
Gapdh	Forward	GCATCTTCTTGTGCAGTGCC
	Reverse	GATGGTGATGGGTTTCCCGT

Abbreviations: RT-qPCR, reverse transcription quantitative polymerase chain reaction; Col1a1, Collagen type I Alpha 1; Runx2, Runt related transcription factor 2; Bmp2, Bone morphogenetic protein 2; Ocn, Osteocalcin; Ripk1, Receptor-interacting serine/threonine-protein kinase 1; Ripk3, Receptor-interacting serine/threonine-protein kinase 3; Mlkl, Mixed lineage kinase domain-like protein; Gapdh, Glyceraldehyde-3-phosphate dehydrogenase.

Supplementary Table 5. Immunohistochemistry antibody information

Antibody	Cat No	Company	Country	Concentration
RIPK1	BS-5805R	Bioss	China	1:100
RIPK3	BS-3551R	Bioss	China	1:150
MLKL	BS-5513R	Bioss	China	1:150
RUNX2	BS-52252R	Bioss	China	1:150
OCN	AF-12303	Abcam	UK	1:200

Abbreviations: RIPK1, Receptor-interacting serine/threonine-protein kinase 1; RIPK3, Receptor-interacting serine/threonine-protein kinase 3; MLKL, Mixed lineage kinase domain-like protein; RUNX2, Runt-related transcription factor 2; OCN, Osteocalcin.

Fig. S4

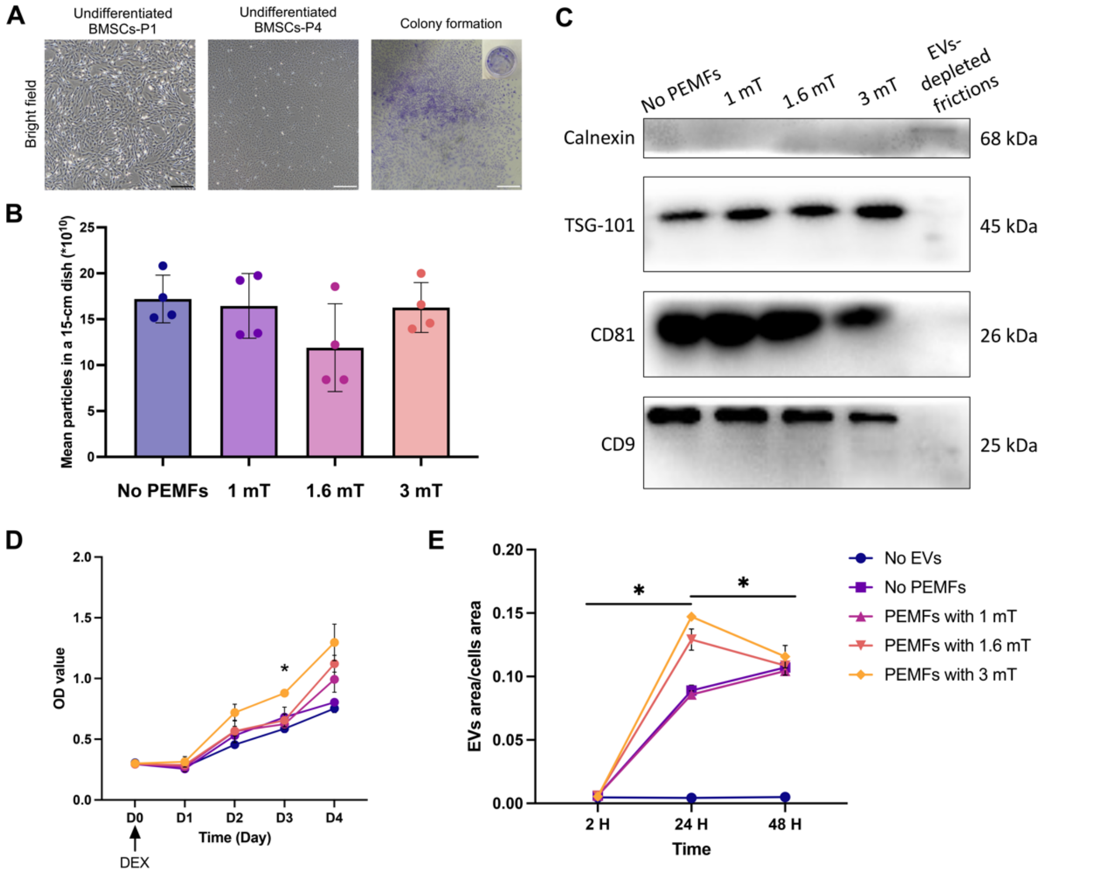

Supplementary Figure S4. Characterization of rat BMSCs and BMSC-EVs under different amplitudes of PEMFs exposure system. (A) Representative images showing the spindle shape at passage one and four, and colony formation ability of BMSCs. Scale bars: 500 μm (white), and 100 μm (black). (B) Protein immunoblots of BMSC-EVs, including three typical positive markers (CD9, CD81, and TSG101) and one negative marker (Calnexin). (B) The number of BMSC-EVs with different parameters of PEMFs (n = 4). Compared with each group as determined by one-way ANOVA and post-hoc analysis. (D) The cell proliferation ability of MC3T3-E1 cells treated with different EVs. Compared to EVs with no PEMFs and 1 mT group, EVs with no PEMFs and 3 mT group enhanced the proliferation, where *$p < 0.05$, ** $p < 0.001$, *** $p < 0.0001$. (E) The ratio of EVs area and MC3T3-E1 cell area treated with EVs from different PEMFs precondition. The ratio at 24 h in EVs with no PEMFs, 1 mT group, 1.6 mT, and 3 mT group significantly higher than 2 h and 48 h, where *$p < 0.05$, ** $p < 0.001$, *** $p < 0.0001$. Compared with each group as determined by two-way ANOVA and post-hoc analysis.

This file includes full-length blots used in the article. The full-length PVDF membrane was cut at 70 and 40 kDa, and we provide a combined full-length blot taken in the bright field. For each original cropped blot, we provide 3 images: one was taken by chemiluminescence, one was taken in the bright field, and the last one was a merged image of the mentioned two.

Fig. S5

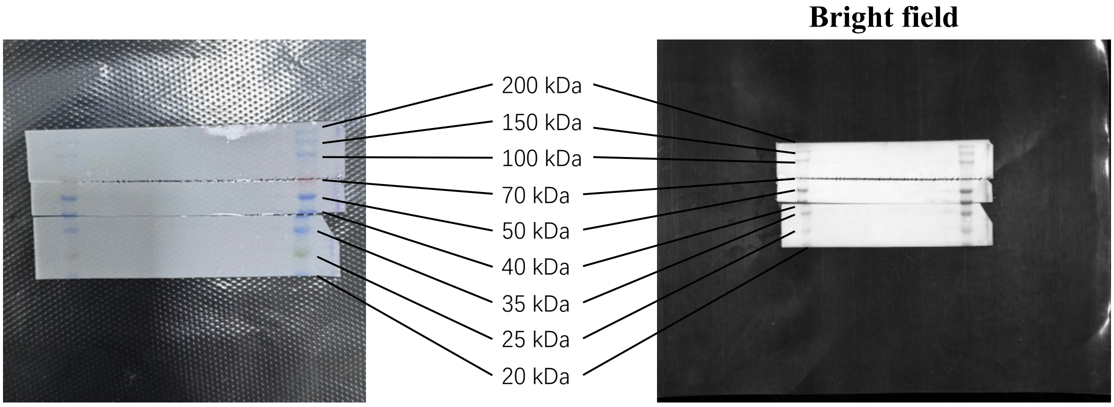

Figure S5. Full-length blots in the bright field of RIPK1, RIPK3, MLKL, and GAPDH in Figure 4.

Fig. S6

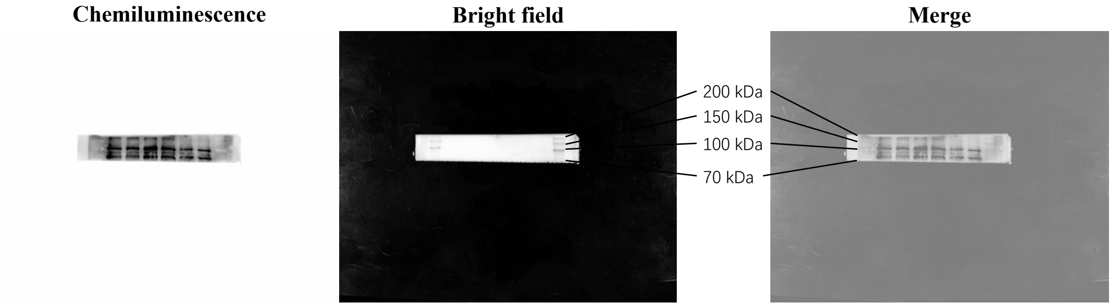

Figure S6. Original blots of RIPK1 in Figure 4 (cut at 70 kDa).

Fig. S7

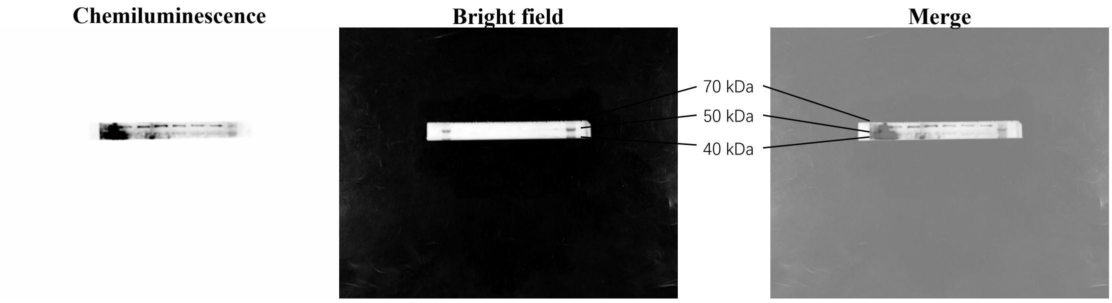

Figure S7. Original blots of MLKL in Figure 4 (cut at 70 kDa and 40 kDa).

Fig. S8

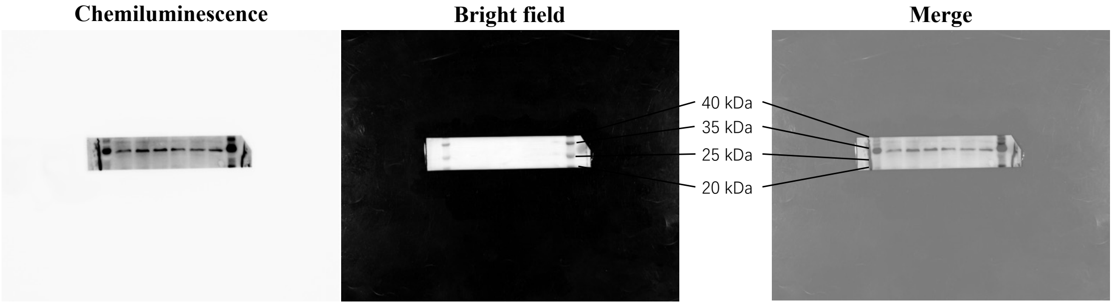

Figure S8. Original blots of RIPK3 in Figure 4 (cut at 40 kDa).

Fig. S9

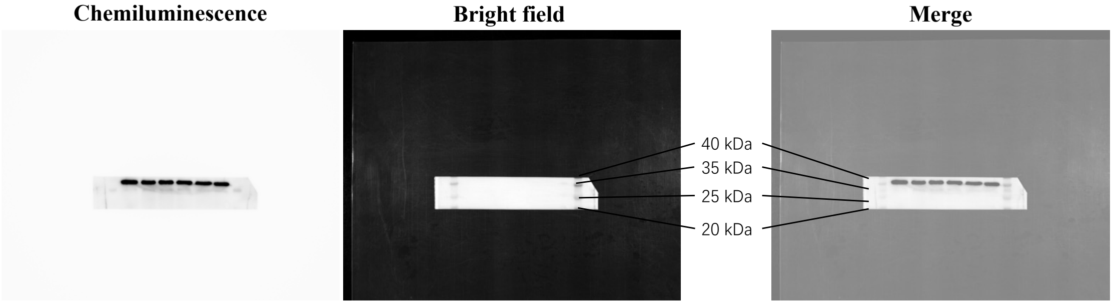

Figure S9. Original blots of GAPDH in Figure 4 (washing with stripping buffer and cut at 40 kDa).
